# Why is it so hard to identify (consistent) predictors of treatment outcome in psychotherapy? – clinical and research perspectives

**DOI:** 10.1186/s40359-023-01238-8

**Published:** 2023-07-05

**Authors:** Silje Elisabeth Hasmo Eilertsen, Thomas Hasmo Eilertsen

**Affiliations:** 1 Haugaland DPS/Department of Research and Innovation, Helse Fonna HF, Haugaland DPS v/ Silje Eilertsen, Postboks 2052, Haugesund, Norway; 2 Stord DPS, Helse Fonna HF, Stord, Norway

**Keywords:** Predictors, Psychotherapy, Anxiety, Depression

## Abstract

**Background:**

Anxiety and depression are two of the most debilitating psychological disorders worldwide today. Fortunately, effective treatments exist. However, a large proportion of patients do not recover from treatment, and many still have symptoms after completing treatment. Numerous studies have tried to identify predictors of treatment outcome. So far, researchers have found few or no consistent predictors applicable to allocate patients to relevant treatment.

**Methods:**

We set out to investigate why it is so hard to identify (consistent) predictors of treatment outcome for psychotherapy in anxiety and depression by reviewing relevant literature.

**Results:**

Four challenges stand out; a) the complexity of human lives, b) sample size and statistical power, c) the complexity of therapist-patient relationships, and d) the lack of consistency in study designs. Together these challenges imply there are a countless number of possible predictors. We also consider ethical implications of predictor research in psychotherapy. Finally, we consider possible solutions, including the use of machine learning, larger samples and more realistic complex predictor models.

**Conclusions:**

Our paper sheds light on why it is so hard to identify consistent predictors of treatment outcome in psychotherapy and suggest ethical implications as well as possible solutions to this problem.

## Introduction

For several decades, psychological research has tried to identify predictors of treatment outcome. More than ten years ago, John Strauss asked “Is Prognosis in the Individual, the Environment, the Disease, or What?” [1]. Two of the most frequent questions asked by patients are “Will I get better from this treatment?”, and ”when?” Do we have the answers?

Depression and anxiety disorders are two of the most common psychological disorders throughout the world today [2]. They are some of the main causes of disability worldwide [3], and untreated the outcomes are poor [4]. Effective treatments exist [e.g. 5–7]. However, a large proportion of patients do not recover from treatment, and many still have symptoms after treatment [8, 9]. Because of this, numerous studies have tried to identify predictors of treatment outcome for psychiatric disorders. A search on Google Scholar for “Predictors of treatment outcome mental disorders” yields more than 2.8 million hits. Limiting this to papers published over the past year still leaves more than 17 000 papers to read. If you spent 15 min reading each paper, it would take you 4250 h, or 177 days of round-the-clock reading just to read the papers published over the past year, indicating a substantial interest in the field. However, even with the considerable effort of trying to identify predictors of treatment outcome, few or none of the suggested predictors predict outcome consistently for anxiety and depression [e.g., 10–17]. For example, some studies find that symptom severity or comorbid depression predict treatment outcome for social phobia and OCD, but far from all [11, 15].

Considering the thousands of predictor studies conducted so far and the lack of consistency in predictors, one could ask whether we should continue researching predictors for psychotherapy outcome at all. On the one hand, one could argue we should continue the search for predictors as treatment choices for each patient based on research is better than “trial and error” or arbitrary conditions (e.g., which therapist was available today?). Looking into this, one should both consider research perspectives and clinical perspectives. By presenting four major challenges each highlighting this problem from different points of view, we intend to positively affect future predictor studies, ultimately improving patient outcomes. These are: Complexity in the nature of the predictors, sample size and statistical power, the complexity of therapist-patient relationship, and difficulties comparing predictor studies. This paper will explore these topics in turn. We will also consider ethical considerations and a few possible solutions. This article will focus mainly on psychotherapy, not pharmacological treatment of psychological disorders.

## Challenge 1: complexity

When exploring what works for whom we investigate predictors, moderators, mediators and interactions. When looking at single predictors, we hope that measures of one variable might foresee measures of another. For instance, we know that patients engaging in cognitive behavioural therapy (CBT) might recover more quickly from depression when compared to a wait list; i.e. receiving treatment or not *predicts* outcome [6]. However, the world is more complicated than this. For instance, we might want to look at moderators, or what works for whom under which circumstances. For example, support from one’s family might *moderate* the effect of internet-CBT for anxiety [18], where in this example patients with more support from the family might profit more from treatment. Furthermore, changes in thoughts and behaviour could *mediate* the relation between CBT and treatment outcome. In other words, it is not therapy in itself, but the changes in thoughts and behaviours following CBT that makes the difference [19]. To make it all even more complicated – let us consider *interactions* between variables: A therapist’s motivation might interact with patients’ engagement to predict outcome in CBT. Patients with low engagement might never succeed and a patient with high engagement always succeed independently of therapist’s motivation. However, if patient engagement is moderate, therapist motivation might make the difference between a poor and a good outcome. Taken together our theoretical modelling of CBT for anxiety or depression will look like this: Changes in thoughts and behaviours, spurred by CBT, influence treatment outcome. However, support from the family moderates this relation, and the interaction between patient engagement and therapist motivation affects the outcome as well. While this seem like a complex study design, it is a quite simple model when compared to the real world. Many predictor studies only look at pre-post designs and might miss the big complex picture.

In addition to the complexity of relations between predictors, one also has to consider the vast number of possible predictors for treatment outcome. Some predictors that might affect treatment outcome for depression are comorbid psychiatric diagnoses [20], comorbid medical conditions [21], personality pathology [22], childhood maltreatment [23], symptom severity and duration [24], sleep disturbances [25] and executive function [26]. There are almost unlimited options considering choices of predictor(s). The predictors could be factors concerning the patient (e.g., personality, motivation), related to the immediate surroundings (e.g., support from family and friends), concerning local society (e.g., access to school or healthcare) or be related to national or global factors (e.g., politics, wars, national financial challenges). How to select the right predictors and how many to investigate simultaneously? When making predictor models, consider the map-and-terrain analogy; when making models of predictions we try to make a simplified map of the world. You do not want your map to have too few details to be useful, but you do not want the map to be as complex as the terrain either - then the map would be useless. You want the sweet balance between explanatory value and reducing complexity.

This brings us to Conclusion 1: Human lives are immensely complex, and there are almost unlimited number of predictors. Furthermore, the relations between different predictors are complex in themselves. This might be one of the reasons for the lack of consistent predictors of treatment outcome in the literature.

## Challenge 2: sample size and statistical power

Sample size determine how many predictors one can investigate – a concept known as “statistical power”. There exist different recommendations for minimum sample sizes for different studies. For example, different models are proposed for continuous vs. binary outcomes [27, 28]. To determine the right sample size required to identify a given statistical difference between groups, researchers use power analyses. Let us look at an example. Say you want to investigate whether comorbid depression affects treatment outcome for social anxiety. You hypothesize a medium effect size (Cohen’s *d* = 0.5) which means you need around 50 patients. However, having thought this through, you take into account the possibility of a smaller effect size (e.g. Cohen’s *d* = 0.3), and the possibility of drop-out when planning the study. Funding is good, ergo you recruit 200 patients with social anxiety that you treat.

If each patient receives 3 h of therapist led evaluations before treatment, it would yield 600 h of work for both therapists and patients. With 15 h of treatment for each patient, you would conduct 3000 h of treatment. Furthermore, you might want patients to fill out forms before and after treatment as well as at follow-up several times. If this takes 4 h for each patient, it would mean 800 more hours of work for the patients. In addition to these theoretical 4400 h of work, the researcher has to prepare the study, plot data, analyze, write the paper, etc. In essence, we are talking about a time-consuming study.

Let us assume that 70% of the patients in the study recover from treatment, meaning 30% do not. In a group of 200 patients, 60 patients do not recover. Let us assume that 50% in the non-recovered group had depression – that would be 30 patients. If only 30% in the recovered group had depression, that would make up 18 patients. In our large resource consuming study, when investigating whether depression affects treatment outcome for social anxiety, we in reality compare these 30 non-recovered vs. the 18 recovered patients. One can hardly assume that 18 patients could be representative of all recovered patients. Even when one factor alone fully explains the lack of response, and the study originally is large enough to detect differences between two groups, the results might not generalize to the population. A recent study indicate there is actually a need of at least 300 patients per treatment arm to be able to precisely select treatment for depression. However, existing recommendations are rarely followed in real life studies [29]. This might be because of the costs associated with large studies as described above. Furthermore, it is important to note that sample size recommendations are not static, or rule-of-thumb based, but will change with new statistical methods, which calls for more sophisticated study designs as the field develops.

Conclusion 2: Many studies have sample sizes too small to identify predictors generalizable to the population. In the words of Lorenzo-Luaces and colleagues (2021): “Personalized medicine and cognitive-behavioral therapies for depression: Small effects, big problems, and [need for] bigger data” [30].

## Challenge 3: the therapeutic relation

There is possibly a wide range of patient factors that could influence treatment outcome. For example, whether the patient wants treatment, whether they are motivated, preference for type of treatment or therapist, hope and life conditions. There are also several therapist factors that might influence treatment outcome. For example, educational level, relational skills, engagement, ability to detect obstacles and change direction in therapy when necessary. Furthermore, there is the multi-faceted concept of therapeutic alliance between therapist and patient, which is hard to define, but important for treatment outcome. Originally defined by Bordin in 1979 [31] as agreement on goals, tasks, and an emotional bond, modern research has expanded on the concept of therapeutic alliance to reveal its vast complexity [32]. For example, matching therapist and patient on specific topics might ensure the therapist has sufficient competence to handle this patient’s particular problems. Constantino et al. (2021) assessed therapist effectiveness on twelve domains (e.g., depression and somatic anxiety) and found evidence that matching (with increasing effects per matched domain) outperformed lack of matching when evaluating patient symptoms and impairment following therapy [33].

Every therapist-patient relation is unique, and there is a complex interaction happening between patient and therapist with many different important aspects [34]. Research has shown relationship between therapist and patient as a whole to play a major role in treatment outcome [e.g., 35]. However, estimates of the therapist effect varies from 0.2 to 29% in different recent studies [36]. This is a multi-faceted area with many methodological difficulties when considering how to operationalize and measure relevant factors. For example, repair of alliance rupture is related to better treatment outcome, but training therapists to repair such ruptures has not been found to predict outcome [37]. One might hypothesize this is because therapists who repair ruptures are warmer, and therefore in reality it is the warmth that contributes to the positive effect, not the repairing. Over the last century there has been a debate considering the conceptualization of working alliance with a wide suggestion of which factors influence the relation between therapist and patient [for a summary, see 38].

Throughout a therapy course, there are often challenges. A clever therapist will address these issues and change direction in the therapy if needed. This indicates that factors that might affect treatment outcome are not static, but dynamic. For example, the timing of interventions might affect outcome [39, 40]. Factors such as motivation, relation between therapist and patient, understanding of material and comorbid diagnoses might change throughout treatment. This illustrates how one should view treatment as a dynamic process with dynamic predictors, in contrast to the idea of only pre-treatment factors predicting treatment outcome.

Conclusion 3: Psychotherapy involves complex interactions between therapist and patient, and every therapeutic relationship is unique. This complexity might make it difficult to identify consistent predictors of treatment outcome.

## Challenge 4: comparing studies

When researchers design predictor studies, they have to ask several important questions. Which therapy to offer? Which study design should I use? What to measure (which predictors)? How to measure them? When to measure? How to operationalize key terms, such as “recovery”?

Different research designs offer different advantages and disadvantages. In randomized controlled studies (RCTs) one has well-controlled variables, but it can be hard to recruit enough participants. An RCT can control for unwanted variability and investigate causality. They are strict and neat. However, considering the limitations it can be hard to know whether the findings can generalize to other settings. In effectiveness studies, on the other hand, it might be easier to recruit and get a more diverse patient group. Hence, you might be able to report on relevant factors, but not be able to report causal relations.

After considering design, the researcher has to decide how to measure effects of treatment. For example, one could administer self-report questionnaires, use structured or semi-structured interviews, therapist evaluations, official recordings, or ask close relatives about their views. Considering questionnaires and interviews there are a large number to choose from, spanning from general measures of psychological distress to specific measures of symptoms and function. A review from 1996 identified more than 1400 different outcome measures for psychological treatments, of which more than half were used just a single time [41]. Furthermore, there are psychometric challenges when using diagnostic psychological instruments. For example, even the SCID-5-CV [42], an interview with good reliability and specificity, have a positive agreement of 73- 97% between conclusion from the interview and clinical diagnoses for different conditions [43]. As if this was not enough, researchers do not always agree on diagnoses of psychiatric disorders. Some researchers rely on the ICD-10 [44], others on DSM-5 criteria [45] and others again on the RDoC [46]. The usage of different measures and different diagnostic criteria links to a big challenge considering validity and reliability of diagnoses in the literature, making it hard to compare different studies.

Another difficulty when comparing different studies is the use of different outcome parameters. Some studies use symptom reduction as indicator of change, some use clinically significant change (CSC), some remission status and a few use side effects or unwanted effects as an indicator of outcome. This is complicated further by relapses, placebo effects, spontaneous remission for some disorders, patient response style and social desirability when filling out forms.

The next consideration in the research design is *when* to measure outcome. Many studies have focused on pre-post designs. However, some studies have investigated predictors while the patient is in treatment, e.g., adherence [47], or predictors from post-treatment to follow-up [48, 49]. Besides, results might differ depending on when you measure outcome, as symptoms and disabilities are unstable over time [50].

Conclusion 4: Different study designs, different measures at different times, disagreement on diagnostic categories and definitions of change make it very difficult to compare results on predictors between studies. While this is not an issue for the individual study, it makes any review or meta-analysis rather difficult. This might be one of the reasons for lack of stable predictors identified by research on treatment outcome in psychiatric disorders. In addition, these are only the difficulties when comparing good studies. Unfortunately, many studies do not even meet current methodological recommendations, making interpretations of findings even harder [51].

## Ethical considerations

There are several ethical challenges considering predictors of treatment outcome in psychotherapy. As of today, we have few consistent predictors of treatment outcome in therapy and therefore do not exclude certain patient groups from treatment. However, if we were to reveal consistent predictors, this might affect whom will receive treatment. Many discrimination challenges might be linked to the identification of predictors. This is especially so if the predictors are unchangeable. We already know that patients more socioeconomic well off might benefit more from treatment [52], but few would argue for excluding patients with less education and hence contributing to «the rich get richer» phenomenon.

There are some predictors that the health care system, health leaders, therapists and patients can change, for instance delivery of evidence-based treatments, engagement, supervision, relational work, communication strategies and tailoring of treatment. Further, there are predictors that the state and society can do something about, for instance economic safety for all families, access to healthcare and training of therapists. However, there are predictors that nobody can change, or that are very difficult to change, such as genetics, gender, age, childhood experiences, ethnicity or sexual orientation. Many predictor studies today focus on such unchangeable predictors [e.g., 53, 54]. One might argue we should more effort put into focusing on factors we can change as this might be more useful, such as managerial support, therapist competence and treatment adherence.

On the other hand, research on non-changeable predictors might have value in helping therapists identify patients at risk and helping them better. One possibility is to take a closer look at treatment response patterns. One study on 834 patients using antidepressants, found groups of patients differing between rapid response after a few weeks, to almost no response even after several months [55]. Patient reported outcome measures could help the therapist identifying these patients earlier. Another recent study from 2021 found patient symptoms might worsen as a consequence of difficulties coping with problems, increasing the risk of treatment failure [56]. Although we cannot prevent patients from facing challenges, by being better informed about predictors of treatment outcome, we might identify patients at risk of unexpected treatment courses. Such patients could receive closer follow-up, more monitoring, advising the therapist of altering the current treatment or allocate the patient to a different, perhaps more suitable treatment alternative.

## Some suggested solutions

There are several possible solutions to the problem of identifying stable predictors of psychotherapeutic outcome. While it is beyond the scope of this article to describe the solutions thoroughly, we will outline a few suggestions. Researchers have employed machine learning hoping to identify consistent predictors of treatment [57]. With machine learning one can handle large amounts of data, and it is data-driven as opposed to theory-driven. Many researchers advocate this as one of the most promising ways forward [e.g., 53, 58]. However, with large amounts of data, the risk of identifying spurious or random correlations is high. In their systematic review and meta-analysis on machine learning, Sajjadian and colleagues recently found a negative relation between study quality and prediction accuracy [57]. They claimed only eight of fifty-four studies had adequate quality. This illustrates how using a new method will not in itself solve all the issues described here, and that it must rather be a part of a multifaceted solution. Even if you identify meaningful correlations, you might end up with “black box algorithms”. I.e., you do not know how and why your models predicts outcome, and risk-among other things-unwanted bias [59].

Another suggested solution is the use of more complex models with multiple predictors to inform treatment recommendations [60]. For example, Jensen and colleagues [61] studied predictors related to different responder status in treatment (acute, sustained responders, slow, continued responders and limited long-term responders). Their results suggest different predictors are related to different response patterns. A second example was presented by Saunders and colleagues [62] investigating a combination of different profiles of predictors in a large-scale study in England. They suggest that a combination of demographic variables and symptom variables might together inform treatment decisions in the future. Together these studies indicate a possible solution to the difficulty of identifying stable single predictors of outcome by building more realistic complex models. Hayes and colleagues [63] advocate this solution and suggest using individual time course data to reveal nonlinear change. However, as most studies are retrospective, there is still the question of whether one can allocate patients to different treatments based on those data. One study where they matched patients with alcohol dependence/abuse with intervention, did not find a better outcome for drinking compared to random assignment of treatment type [64]. However, one recent study found promising results matching patients with anxiety and depression to therapy through the tool “Link-me” in Australia [65]. A second recent promising approach using prospective research to link patients to low vs. high intensity treatment is “The Leeds Risk Index” [66]. A third promising approach is the use of the Personalized Advantage Index (PAI), a complex personalized model to predict optimal treatment for the individual in question, and to match patients with treatment options [e.g., 67, 68, 69]. However, a recent external validation study of the PAI found mixed results when applying PAI in two samples to allocate patients to treatment in the Netherlands [70]. This is to our knowledge the first study cross-trial validating predictions retrospectively. There is a need for more research to explore and validate tools to link patients to specific treatments.

A third suggested solution is the use of multicenter studies where several research environments contribute to large samples. At face value, this increases the generalizability of the results, mentioned in challenge 4, and increases sample size, addressing challenge 2. However, multicenter studies also means more context variables, thus more complexity, consequently increasing the problems described in challenge 1. Furthermore, while multicentre studies might split cost between several research teams, they do not solve the cost issue of large samples, and they often require increased logistic work.

To overcome the difficulties of sample sizes, we suggest multicenter studies even though this comes with hassles as described above. There is a need for more replication studies in the literature in order to be able to compare studies more easily. It is also advisable to implement quality assurance as an integrated part of treatment in the health care system to provide useful hands-on information about treatment results and needs for improvement on treatment as usual. Furthermore, as mentioned at the very beginning of this paper – we have solid evidence based treatments for anxiety and depression. The first step to provide good health care for patients is to implement these evidence-based treatments, offer solid therapist training and ensure quality in services for the population.

## Conclusions

In this paper, we investigated the lack of consistent predictors for outcome in psychological treatment for anxiety and depression. We suggest four challenges; (a) the complexity of human lives, (b) sample size and statistical power, (c) the complexity of therapist-patient relationships, and (d) the lack of consistency in study designs, which together might explain the lack of consistency in predictor research. Possible solutions include the use of machine learning, more complex predictor models, multicenter studies with larger samples, increase the usage of replication studies and implementing quality assurance in the health care systems.

## Data Availability

Not applicable.
